# Follicle-stimulating hormone is negatively associated with nonalcoholic fatty liver disease in a Chinese elderly population: a retrospective observational study

**DOI:** 10.1186/s12902-023-01427-x

**Published:** 2023-08-07

**Authors:** Xiaoming Li, Ning Xin, Tailin Guo, Ziyu Wu, Ying Zheng, Lan Lin, Qianwen Li, Fan Lin

**Affiliations:** 1 Department of Geriatric Medicine, Fujian Provincial Key Laboratory of Geriatric Diseases, Fujian Medical University, Fujian Provincial Hospital, Fujian Provincial Institute of Clinical Geriatrics, Fuzhou, 350001 China; 2 Key Laboratory of Medical Big Data Project of Fujian Province, Fujian Medical University, Fujian Provincial Hospital, Fuzhou, 350001 China

**Keywords:** Nonalcoholic fatty liver disease, Follicle-stimulating hormone, Elderly, Men, Women

## Abstract

**Background:**

Several studies have explored the connection between follicle-stimulating hormone (FSH) and nonalcoholic fatty liver disease (NAFLD). However, the impact of FSH elevation on NAFLD remains a topic of debate. Hence, this investigation aimed to evaluate the potential correlation between FSH levels and NAFLD in the aging population.

**Methods:**

This was a retrospective observational cross-sectional study between July 2017 and August 2018 in our hospital. We used data obtained from 455 patients over 60 years old. Anthropometrics and laboratory tests were performed for each patient. NAFLD was diagnosed by sonographic features and the fatty liver index (LFI).

**Results:**

Of the 455 patients, 200 (43.96%) had NAFLD on their ultrasound and 169 (37.14%) had NAFLD according to the LFI. An intraclass correlation coefficient of the two methods was 80.4% (*P* < 0.001). People with NAFLD on their ultrasound showed lower FSH levels (52.68 vs. 61.39 IU/L) and more unfavorable metabolic profiles. FSH was negatively correlated with age, alanine aminotransferase, estradiol, testosterone, systolic blood pressure, waist, body mass index, fasting blood glucose, postload plasma glucose and positive associated with total cholesterol, high-density lipoprotein-cholesterol and low-density lipoprotein-cholesterol by Spearman correlation analysis (all *P* < 0.05). By controlling for all confounding factors, the odds ratios (OR) of FSH for NAFLD were determined in elderly individuals, both men and women, aged 60–70 years and over 70 years. These ORs were found to be 0.937, 0.982, 0.983, and 0.973, respectively, with corresponding 95% confidence intervals (CI) of 0.892–0.984 (*P* = 0.009), 0.971–0.993 (*P* = 0.002), 0.967–0.999 (*P* = 0.033), and 0.958–0.989 (*P* = 0.001). In addition, our findings demonstrated no significant correlation between FSH and advanced fibrosis when adjusting for potential covariates. The OR for advanced fibrosis was 0.979 (95% CI, 0.938–1.022, *P* = 0.339). Additionally, ROC curve analysis showed an optimal cut-off value of 66.91 for women and 15.25 for men for NAFLD diagnosis.

**Conclusions:**

There was an inverse relationship observed between levels of FSH in the blood serum and NAFLD in the elderly population. These findings suggest that reduced FSH levels might serve as a potential risk factor or biomarker for NAFLD in the elderly.

**Supplementary Information:**

The online version contains supplementary material available at 10.1186/s12902-023-01427-x.

## Introduction

Nonalcoholic fatty liver disease (NAFLD) is a broad term encompassing a spectrum of liver disorders, including fatty liver as well as nonalcoholic steatohepatitis, with the presence or absence of inflammation and fibrosis [1, 2]. The prevalence of NAFLD ranges from around 24–35% among the general population across different nations, and its increasing occurrence poses a significant public health challenge. With its high prevalence and growing association with the burden of advanced liver disease, NAFLD demands attention in the field of public health [3, 4]. Many studies reported that NAFLD is associated with a range of metabolic disorders, including but not limited to type 2 diabetes mellitus (T2DM), obesity, dyslipidemia and metabolic syndrome (MS). Moreover, substantial evidence indicates that individuals with NAFLD may exhibit a slightly elevated risk of mortality in comparison to the general population, regardless of conventional risk factors [3, 5–7]. Hence, the investigation of the underlying mechanism governing the regulation of NAFLD is highly intriguing.

The etiology of NAFLD involves multiple factors [8, 9]. There is growing evidence that sex steroids are involved in the pathogenesis of NAFLD [10]. Prior studies reported that testosterone prevents hepatic steatosis in men [11, 12], while estradiol exhibits analogous protective effects against liver steatosis in both males and females [10, 13]. However, the association between follicle-stimulating hormone (FSH) and NAFLD is quite uncommon. FSH, also known as a gonadotropin due to its role in stimulating the gonads, is synthesized by the anterior pituitary gland in the brain [14]. Initially, FSH was primarily perceived to impact the reproductive system; however, subsequent investigations revealed its influence on non-reproductive organs and tissues as well. These effects encompass the promotion of lipid biosynthesis [15], increased susceptibility to osteoporosis [16], and proliferation of cholangiocytes in mice [17]. Notably, numerous studies have provided insights into the significance of FSH signaling in the regulation of hepatic lipid [18, 19] and glucose metabolism [20] through both experimental and clinical investigations. Several studies have investigated the correlation between FSH and NAFLD. Nevertheless, the impact of elevated FSH levels on NAFLD remains a topic of debate. A study conducted on postmenopausal women revealed an association between lower FSH levels and an increased risk of NAFLD [21]. In contrast, a separate investigation involving Chinese men over the age of 80 reported lower FSH levels being linked to a reduced risk of NAFLD [22]. These conflicting findings contribute to the ongoing controversy surrounding the influence of FSH on NAFLD.

Therefore, our objective was to evaluate the potential correlation between FSH levels and NAFLD in the elderly population including men and women over 60, who have elevated FSH and low levels of testosterone and estrogen. And further explore the relationship between FSH and advanced fibrosis in individuals diagnosed with NAFLD.

## Materials and methods

### Study design and patient population

We performed a retrospective observational cross-sectional study between July 2017 and August 2018 at our hospital. The participants of this study were patients who visited the hospital for health checkups with examination of ultrasound of liver and sex hormone test. A total of 726 patients who reported alcohol consumption lower than 140 g per week underwent initial screening, and 271 of them were subsequently excluded based on the following reasons: sex hormone related tumors (breast cancer, prostate cancer and testicular cancer) and various kinds of advanced tumors (n = 136), pituitary tumor (n = 16), hypopituitarism (n = 9), hypothyroidism, subclinical hypothyroidism (n = 37), hyperthyroidism and subclinical hyperthyroidism (n = 22), adrenocortical dysfunction (n = 8), the utilization of medications with the potential to influence sex hormone levels (e.g., spironolactone, antiparkinsonian drugs, and antipsychotic drugs) (n = 10), hepatitis (hepatitis B surface and hepatitis C antibody), liver dysfunction (n = 21) and renal insufficiency (n = 12). Finally, four hundred and fifty-five subjects were included in the final analyses.

The Ethics Committee of Fujian Provincial Hospital granted approval for this study. Given its retrospective nature, the requirement of informed consent was waived by the Ethics Committee of Fujian Provincial Hospital. All procedures followed relevant guidelines and regulations, ensuring compliance with the Declaration of Helsinki for research involving human participants, human material, or human data.

### Anthropometric and biochemical measurements

Weights and heights were recorded in participants wearing lightweight gowns. Waist circumferences were measured in a standing position, at the midpoint between the lowest rib margin and the iliac crest. Body mass index (BMI) was computed by dividing the weight by the square of the height (kg/m^2^) [23]. Systolic blood pressure (BP) was assessed within the initial 72 h of admission, with participants lying down and using a standard protocol for measurement, utilizing a mercury sphygmomanometer.

Blood samples were collected from non-diabetic individuals before and 2 h after the administration of a 75-g oral glucose challenge to measure venous glucose levels. Enzymatic analysis was performed to measure fasting blood glucose (FBG), postload plasma glucose (PPG), total cholesterol, high-density lipoprotein-cholesterol (HDL-c), low-density lipoprotein-cholesterol (LDL-c), triglycerides, and liver enzymes. FSH, testosterone and estradiol were determined by radioimmunoassay. Venous blood samples were obtained from all patients during the morning hours of 8:00 to 11:00 AM, following a minimum 8-hour overnight fasting period.

### Ultrasonographic examination

All patients underwent hepatic ultrasonography scans conducted by an experienced radiologist, who was unaware of participants’ information. Diagnosis of hepatic steatosis was established by identifying characteristic sonographic features, such as reduced visibility of intra-hepatic vessel borders and diaphragm, evidence of ultrasound beam attenuation, and diffuse liver hyper-echogenicity compared to the kidneys [24].

### Definitions and diagnostic criteria

The definition of hypertension was determined according to the criteria specified in the Seventh Report of the Joint National Committee on Prevention, Detection, Evaluation, and Treatment of High Blood Pressure [25]. DM was based on the 2020 guidelines set forth by the American Diabetes Association [26]. The definition of MS was based on a joint interim report [27]. We also used the FLI as a predictor of hepatic steatosis. FLI is calculated as: FLI = (e ^0.953*loge (triglycerides)^ + 0.139*BMI + 0.718*loge (gamma glutamyl-transferase (GGT)) + 0.053*waist circumference − 15.745) / (1 + e^0.953*loge (triglycerides)^ + 0.139*BMI + 0.718*loge (GGT) + 0.053*waist circumference − 15.745) * 100. A FLI < 30 rules out fatty liver [28]. The evaluation of liver fibrosis was performed using the FIB-4 index, which was derived from a specific formula: age [years] × AST [U/L] / (platelet [10^9^/L] × (ALT [U/L])^1/2^). FIB-4 index > 2.67 was used as the threshold to define advanced fibrosis [29].

### Statistical analysis

Continuous variables were presented as mean ± standard deviation, while categorical variables were expressed as numbers with proportions. Skewed variables, such as alanine aminotransferase (ALT), HDL-c, triglycerides, FSH and testosterone, underwent logarithmic transformation to enhance normality before analysis. To assess significant differences in frequency distributions and means, Chi-squared and Student’s t-tests were employed, stratifying the participants based on the presence or absence of NAFLD and gender. The association between FSH and metabolic factors was evaluated using Spearman’s rank-order correlation analysis. The findings were presented as Spearman’s rank correlation coefficient. To further detect the FSH between NAFLD on their ultrasound, logistic regression models were employed. These models adjusted for potential confounders by stratifying according to gender and age. The original model was not adjusted. The first model was adjusted for age, gender, BMI, systolic BP, FBG, triglycerides, total cholesterol, HDL-c, LDL-c, ALT, AST, estradiol and testosterone. The second model included age, gender, ALT, AST, estradiol, testosterone, DM, dyslipidemia, obesity and hypertension. All variables except gender were included in the models when analyses were applied for the men and women. All logistic regression models were also applied to identify the correlation between FSH and NAFLD as defined by LFI criteria. To explore the potential correlation between FSH and advanced fibrosis, a Spearman correlation analysis was conducted between FSH and the FIB-4 index. Subsequently, a multi-adjusted binary logistic regression model was utilized, with adjustments made for age, alcohol usage, diabetes, hyperlipidemia, estradiol and T, all of which are known to be associated with hepatic fibrosis. The findings were reported as odds ratios (ORs) along with their corresponding 95% confidence intervals (CIs). There is no collinearity between all variables during analysis. The study utilized receiver operating characteristic (ROC) curve analysis to determine the ideal threshold value of FSH in the identification of individuals with NAFLD. The cut-off value was determined by utilizing the Youden index. Statistical analysis was performed using SPSS 17.0 for Windows (SPSS 17.0 Inc., USA). All tests were two-tailed, and significance was set at a *P*-value less than 0.05.

## Results

### Characteristics of the study population

Table 1 provided an overview of the key characteristics observed among the study population. Of the 455 patients who were enrolled in this study, 200 (43.96%) showed hepatic steatosis on ultrasound findings and 169 (37.14%) showed hepatic steatosis according to the fatty liver index. An intraclass correlation coefficient of the two methods was 80.4% (*P* < 0.001). The average age of the patients was 71.44 ± 8.80 years, whereas the mean BMI was 24.06 ± 3.62 kg/m^2^. DM, prediabetes, hypertension, dyslipidemia, obesity and MS were identified in 50.33%, 6.4%, 64.18%, 66.08%, 62.64% and 74.51% of the subjects, respectively. Table 1 also presented the baseline characteristics of patients, categorized based on their NAFLD status. People with NAFLD showed lower FSH levels (52.68 vs. 61.39 IU/L) and more unfavorable metabolic profiles. Individuals with hepatic steatosis exhibited significantly elevated SBP, waist circumference, BMI, FBG, ALT and triglyceride levels, while demonstrating lower levels of HDL-c and testosterone, as compared to those without liver fat infiltration (all *P* < 0.05). Patients with NAFLD had a higher frequency of dyslipidemia, hypertension, obesity, DM and MS than patients without NAFLD (all *P* < 0.05). There were no significant differences observed between the two groups in terms of age, sex, current smoking, diastolic BP, PPG, AST, total cholesterol, LDL-c and estradiol levels.

**Table 1:** Characteristics of all the study participants categorized by the presence of NAFLD on ultrasound

	ALL	Non-NAFLD	NAFLD	*P value*
	N = 455	(n = 255)	(n = 200)	
Demography				
Age(ys)	71.44 ± 8.80	71.39 ± 8.38	71.51 ± 9.33	0.888
Men(%)	21.5%(98)	24.3%(62)	18%(36)	0.109
Current smoker(%)	9.67% (44)	11% (28)	8% (16)	0.292
systolic BP (mmHg)	136.49 ± 20.17	133.76 ± 19.20	139.75 ± 20.87	0.005
diastolic BP (mmHg)	74.06 ± 10.79	73.79 ± 11.54	74.38 ± 9.85	0.602
Fat-related				
Waist (cm)	82.82 ± 7.96	82.82 ± 7.96	87.73 ± 12.64	0.003
BMI	24.06 ± 3.62	22.34 ± 3.04	25.62 ± 3.40	< 0.001
Glucose-related				
FBG (mmol/L)	6.92 ± 2.88	6.38 ± 2.47	7.59 ± 3.2	< 0.001
PPG (mmol/L)	9.25 ± 4.46	9.22 ± 5.04	9.27 ± 3.96	0.954
Liver Enzyme-related (IU/L)				
ALT^†^	21.80 ± 13.64	19.79 ± 12.12	24.36 ± 14.20	< 0.001
AST	21.48 ± 10.58	22.20 ± 10.02	22.85 ± 11.27	0.512
Lipid-related				
TC (mmol/L)	4.75 ± 1.13	4.74 ± 1.16	4.76 ± 1.08	0.895
HDL-c(mmol/L) ^†^	1.27 ± 0.41	1.36 ± 0.44	1.15 ± 0.33	< 0.001
LDL-c(mmol/L)	3.02 ± 1.01	2.99 ± 1.05	3.08 ± 0.96	0.347
triglycerides (mmol/L) ^†^	1.59 ± 1.19	1.33 ± 1.03	1.93 ± 1.29	< 0.001
SEX hormone FSH^†^ (IU/L)	54.63 ± 31.35	61.39 ± 33.61	52.68 ± 27.01	0.004
estradiol (pg/ml)	31.19 ± 22.62	30.66 ± 21.45	28.53 ± 20.52	0.285
testosterone^†^ (nmol/L)	3.59 ± 5.41	4.34 ± 6.23	2.76 ± 4.19	0.027
Syndrome-related(%)				
Dyslipidemia	66.08%(301)	61. 2% (156)	72.5% (145)	0.001
Hypertension	64.18%(292)	57.6% (147)	72.5% (145)	< 0.001
Obesity	62.64% (285)	48.2% (123)	81.0% (162)	< 0.001
DM related(%)				< 0.001
NGT	43.30%(197)	52.9%(135)	31%(62)	
IGT	6.4%(29)	3.9%(10)	9.5%(19)	
DM	50.33%(229)	43.1%(110)	59.5%(119)	
Metabolic syndrome	74.51%(339)	64.7%(165)	87% (174)	< 0.001

Data were expressed as the means ± SD, frequencies

^†^Logarithmically transformed when compared

NAFLD: nonalcoholic fatty liver disease; BP: blood pressure; BMI: body mass index; FBG: fasting blood glucose; PPG: postload plasma glucose; ALT: alanine aminotransferase; AST: aspartate aminotransferase; TC: total cholesterol; HDL-c: high-density lipoprotein cholesterol; LDL-c: low-density lipoprotein cholesterol; FSH: follicle-stimulating hormone; NGT: normal glucose tolerance; IGT: impaired glucose tolerance; DM: diabetes mellitus

Table 2 showed the FSH and sex hormone levels of patients stratified by gender. Men showed lower FSH levels (22.70 vs. 65.27 IU/L) and higher estradiol (49.15 vs. 25.21 pg/ml) and testosterone (11.36 vs. 1 nmol/L) level compared with women (all *P* value < 0.001).

**Table 2:** Sex hormone levels categorized by gender

	Men	Women	*P* value
	N = 98	N = 357	
FSH^†^ (IU/L)	22.70 ± 18.84	65.27 ± 27.20	< 0.001
estradiol (pg/ml)	49.15 ± 26.23	25.21 ± 17.66	< 0.001
testosterone^†^ (nmol/L)	11.36 ± 5.95	1.00 ± 0.66	< 0.001

FSH: follicle-stimulating hormone;

^†^Logarithmically transformed when compared

### Association of FSH with NAFLD

The findings from the Spearman correlation analysis between FSH and various metabolic factors in all participants are summarized in Table 3. Age, systolic BP, waist circumference, BMI, FBG, PPG, ALT, estradiol and testosterone showed a negative correlation with FSH, while total cholesterol, HDL-c and LDL-c demonstrated a positive association (all *P* < 0.05).

**Table 3:** Spearman correlation of FSH with metabolic factors in all subjects

	FSH	*P*
Age	-0.187	< 0.001
systolic BP	-0.120	0.022
diastolic BP	-0.038	0.478
Waist	-0.258	0.001
BMI	-0.207	0.005
FBG	-0.354	< 0.001
PPG	-0.482	< 0.001
ALT	-0.140	0.003
AST	-0.070	0.137
total cholesterol	0.252	< 0.001
HDL-c	0.262	< 0.001
LDL-c	0.205	< 0.001
triglycerides	-0.018	0.699
estradiol	-0.354	< 0.001
testosterone	-0.482	< 0.001

FSH: follicle-stimulating hormone; BP: blood pressure; BMI: body mass index; FBG: fasting blood glucose; PPG: postload plasma glucose; ALT: alanine aminotransferase; AST: aspartate aminotransferase; HDL-c: high-density lipoprotein cholesterol; LDL-c: low-density lipoprotein cholesterol

In order to explore the relationship between FSH and NAFLD, logistic regression models were utilized to estimate ORs, both with and without adjustment for confounding factors. In a univariate logistic regression analysis for all subjects, a significant inverse association was observed between FSH and NAFLD. After adjusting for all confounding factors, ORs of FSH for NAFLD were 0.981 and 0.979, respectively (Model 1: 95%CI, 0.969–0.993, *P* = 0.002 and Model 2: 95%CI, 0.968–0.989, *P* < 0.001, respectively, Table 4).

**Table 4:** Logistic regression analysis of the relationship between NAFLD on ultrasound and FSH according to gender and age stratification

			adjusted association	
		Unadjusted association	Model 1	Model 2
All	Odds ratios	0.991	0.981	0.979
	95%CI	0.985–0.997	0.969–0.993	0.968–0.989
	*P* value	0.003	0.002	< 0.001
Men* N = 98	Odds ratios	0.971	0.859	0.937
	95%CI	0.942-1.000	0.764–0.966	0.892–0.984
	*P* value	0.046	0.011	0.009
Women* N = 357	Odds ratios	0.981	0.980	0.982
	95%CI	0.973–0.990	0.967–0.993	0.971–0.993
	*P* value	< 0.001	0.003	0.002
Age stratification				
between 60–70 years	Odds ratios	0.988	0.986	0.983
N = 230	95%CI	0.979–0.996	0.969–1.003	0.967–0.999
	*P* value	0.005	0.114	0.033
Over 70 years	Odds ratios	0.993	0.963	0.973
N = 225	95%CI	0.985–1.002	0.943–0.984	0.958–0.989
	*P* value	0.150	0.001	0.001

Model 1 included age, gender, body mass index, systolic blood pressure, fasting blood glucose, triglycerides, total cholesterol, high-density lipoprotein cholesterol, low-density lipoprotein cholesterol, alanine aminotransferase, aspartate aminotransferase, estradiol and testosterone for adjustment

Model 2 included age, gender, alanine aminotransferase, aspartate aminotransferase, estradiol, testosterone, diabetes mellitus, dyslipidemia, obesity and hypertension for adjustment

NAFLD: nonalcoholic fatty liver disease; FSH: follicle-stimulating hormone

*: All variables except gender were included in the models

To gain a better understanding of the sexual disparities underlying the disease, we continue to conduct sex-specific analyses. As shown in Table 4, we also found an inverse association between serum FSH levels and NAFLD for men and women in all models (all *P* < 0.05). Following the adjustment for all potential confounding factors, OR of FSH for NAFLD in elderly men was 0.937 (95%CI, 0.892–0.984, *P* = 0.009). Likewise, women had the similar result after adjusting for all confounding factors (OR, 0.982, 95% CI, 0.971–0.993, *P* = 0.002, shown in Table 4).

To explore the association between FSH and NAFLD in different age groups, we divided the people into two groups based on age: individuals aged between 60 and 70 years (N = 230) and those above 70 years (N = 225). We found that ORs of FSH for NAFLD in people age between 60 and 70 years and over 70 years old were 0.983 and 0.973 after adjusting for all confounding factors, respectively (95%CI, 0.967–0.999, *P* = 0.033 and 0.958–0.989, *P* = 0.001, respectively) (shown in Table 4). Logistic regression models were also employed to detect the relationship between FSH and NAFLD defined by LFI adjust for potential confounders according to gender and age stratification. The results were similar to the founding of FSH and NAFLD defined by ultrasound (shown in Supplement 5).

Through additional investigation into the correlation between FSH and advanced fibrosis in patients diagnosed with NAFLD (N = 200), we observed no significant correlation between FSH and the FIB-4 index (r = -0.042, *P* = 0.553). Even though adjustments were made for multiple variables such as age, BMI, alcohol usage, diabetes, hyperlipidemia, estradiol, and T, the link between FSH and advanced fibrosis remained statistically non-significant. The OR for advanced fibrosis was calculated to be 0.979 (95% CI, 0.938–1.022, *P* = 0.339).

### ROC curve analysis of the utility of FSH in the prediction of NAFLD

**Fig. 1:**
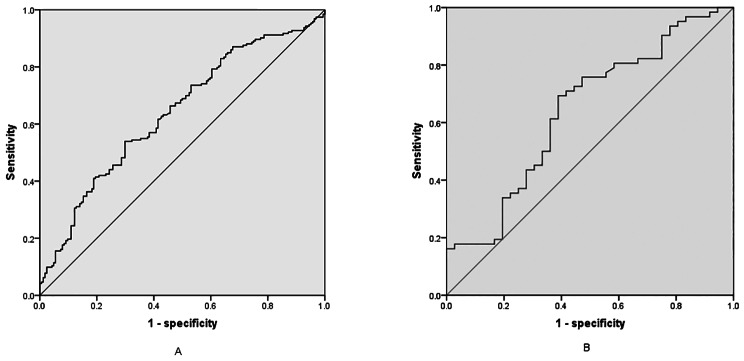
Receiver operating characteristic (ROC) curve evaluating the utility of FSH in the diagnosis of NAFLD in elderly women (Fig. 1A) and men (Fig. 1B). The area under the curve was 0.639 for women (95%CI, 0.582–0.696, *P* = 0) and 0.640 for men (95%CI, 0.525–0.756, *P* = 0.021) respectively

The ability of FSH to predict NAFLD was evaluated by ROC curve analysis for women and men (Fig. 1). The area under the curve was 0.639 for women (95%CI, 0.582–0.696, *P <* 0.001) and 0.640 for men (95%CI, 0.525–0.756, *P* = 0.021) respectively. The optimal cut-off value of FSH for the diagnosis of NAFLD was 66.91 for women and 15.25 for men. The sensitivity and specificity of FSH threshold value for diagnosing NAFLD was 53.9% and 70.1% for women and 69.4% and 61.1% for men, respectively.

## Discussion

In this study, we discovered an independent inverse association between FSH and NAFLD in the elderly Chinese population, characterized by a generally consistent hormonal profile displaying increased serum FSH levels and decreased serum androgens and estrogens. Importantly, our results were not attributable to body fatness, estradiol or testosterone concentrations, or the presence of common metabolic disorders. Furthermore, we determined that the optimal FSH threshold for diagnosing NAFLD was 66.91 for females and 15.25 for males.

The present study provided compelling evidence of a notable correlation between FSH and the components of metabolic syndrome. It was consistent with previous studies. Our preliminary analysis found that FSH was inversely associated with FBG and PPG. And previous investigation also demonstrated a significant link between reduced FSH levels and the presence of diabetes [20]. Moreover, we found that lipid level was positively associated with FSH. The findings are consistent with other research, supporting the involvement of FSH in lipid biosynthesis [15] as well as its potential interaction with hepatocyte receptors, leading to the reduction of LDL receptor levels and consequent elevation of circulating LDL-c levels [18]. Increasing evidence is indicating the potential metabolic function of FSH. Furthermore, NAFLD is widely recognized as a hepatic manifestation of MS [30]. Thus, the observed correlation between FSH and NAFLD in our study not only provides additional evidence but also contributes to the emerging understanding of the novel role of FSH in metabolic disorders.

A significant finding of this study is the independent reverse correlation between FSH and NAFLD in older adults, regardless of potential confounding factors. Limited research has been conducted on the impact of FSH on NAFLD in humans, and the findings have been inconclusive. In our previous investigation, we discovered an independent correlation between the diurnal rhythm of FSH and NAFLD in an older population. However, given the constraint of a limited sample size, we were unable to establish a conclusive association between FSH and NAFLD [31]. This relationship in postmenopausal women was investigated in another study, which also found a negative correlation. And they did not examine the correlation in men [21, 32]. However, a different study revealed a positive association between FSH levels and the susceptibility to NAFLD in elderly males aged 80 years and above [22]. The reasons behind variations in research outcomes regarding the association between FSH and NAFLD remain uncertain, although age might exert a certain degree of influence on these disparities. The data suggested that inverse correlations between FSH and NAFLD were found in postmenopausal women (mean age was 69.91 in our finding and 64.27 in Wang’s finding) and relatively young men in our finding (mean age 77 years with only 39 men over 80 years). In contrast, positive association with NAFLD was found in older men (mean age 85.5). As a result, variations in age could potentially yield disparities in outcomes. Another possible reason for the discrepancies may include a shifting correlation between FSH isoforms and age, which is supported by the literature. Age-related alterations in the distribution of isoforms, combined with inter-individual variability, can influence the biological effects of FSH [33]. Investigating potential variations in the functional role of FSH in relation to age represents a crucial and promising avenue for future research endeavors.

The precise mechanisms underlying the correlation between FSH and NAFLD remain poorly understood. Abdominal obesity emerges as a potential explanation for this association. Previous studies have highlighted a noteworthy finding that links obesity with the dampening effect on the elevation of FSH following the final menstrual period [34]. Additionally, another study discovered that weight reduction resulted in an elevation in FSH levels among postmenopausal women who were overweight or obese [35]. And it is generally known that abdominal obesity is involved in the pathogenesis of NAFLD. Therefore, it is plausible to deduce that the decrease in FSH levels in individuals with NAFLD may be ascribed to a greater incidence of obesity. However, the significant association between FSH and NAFLD persisted after adjusting for BMI. This discovery suggested that the correlation between FSH and NAFLD was unaffected by the adiposity. Another possibility is that FSH may regulate liver triglyceride metabolism by binding to FSH receptors (FSHR) on liver cells. Because FSHR is also expressed on hepatocytes, regulating cholesterol anabolism [18]. FSH is known to modulate lipid metabolism in adipose tissue through various mechanisms. For instance, it can bind to Gas-coupled FSHR, initiating the classical cAMP/PKA/CREB pathway and promoting the production of lipids as well as lipogenic factors [15]. Additional investigations have revealed that FSH binds to Gαi-coupled FSHR, activating Ca^2+^ channels and triggering intracellular calcium influx. This influx, in turn, stimulates the formation of lipids through activation of the CREB pathway [36]. FSH also aids in enhancing FSH secretion through GnRH by phosphorylating PTPN5 [37]. However, it is crucial to acknowledge that these effects have, to date, exclusively been verified in adipose tissue. It can be speculated that FSH may regulate triglyceride metabolism in hepatocytes through opposing pathways. FSH could potentially inhibit the adipogenesis signaling pathway and prevent hepatic steatosis by binding to FSHR on liver cells and downregulating the classic cAMP/PKA/CREB pathway or CREB pathway. A recent study has revealed a third possible mechanism by which FSH affects liver lipid metabolism. This study revealed that the paracrine action of FSH on pituitary corticotropes can serve as a mechanism to inhibit the synthesis of corticosterone and protect against hepatic steatosis [38]. The fourth possible reason may be associated with inhibin A. Previous research has revealed a positive correlation between FSH levels and the concurrent rise in activin A concentrations [39, 40]. Recent research has demonstrated that Activin A exhibits anti-inflammatory characteristics and increase insulin resistance [41]. Therefore, increased FSH is associated with increased activin A, which may decrease the prevalence of NAFLD. The fifth potential explanation could be attributed to FSH’s impact on adipose tissue. Research indicates that FSH can induce an excessive synthesis of lipids in adipose tissue, potentially resulting in the transfer of lipids from the liver to adipocytes, particularly in aging populations [36]. Thus, we suppose that the redistribution of fat accumulation regulated by FSH during aging of men and women leads to the improvement of lipid accumulation in liver. However, further investigation is required to elucidate the underlying mechanism.

In this study, we found that the use of FLI for fatty liver is consistent with ultrasound diagnosis for fatty liver (intraclass correlation coefficient 80.4%). The result shows that FLI is an easy and relatively accurate method to diagnose hepatic steatosis. It is an option for diagnosing NAFLD, especially for large-scale epidemiological investigations in clinic practice.

No correlation was observed between advanced fibrosis and FSH in our study. It’s in line with a previous investigation [22] that explored the link between serum FSH levels and NAFLD in elderly Chinese males above 80 years of age. The two studies focus on the elderly population in China, characterized by a relatively stable endocrine profile featuring elevated levels of FSH in the serum and decreased levels of both androgens and estrogens. The reasons for negative results may include the following aspects. Firstly, FSH may not be involved in the progression of advanced fibrosis of NAFLD, as there is still no evidence to support this viewpoint. Secondly, a relatively small sample size may also lead to negative results. There were 200 people with NAFLD in our study, of whom 19 were considered to have advanced fibrosis. Similarly, there were 108 NAFLD patients in their study and the subset of patients presenting with advanced fibrosis may be even more limited in size. Therefore, a relatively small sample size may be also a reason for it. Finally, hepatic fibrosis was assessed using the FIB-4 index without histological confirmation. Additional investigations are required to assess the suitability of the suggested threshold (FIB-4 > 2.67) for diagnosing advanced fibrosis in the elderly population. To validate this finding, it is imperative to conduct larger prospective studies.

Within our investigation, we have successfully determined the optimal cut-off values for FSH in diagnosing NAFLD. For elderly women, a FSH cut-off value of < 66.91 indicates a higher risk of NAFLD, whereas for men, a cut-off value of < 15.25 is indicative of increased susceptibility to NAFLD. The higher value of FSH found in women may reflect lower level of estradiol and testosterone in them. While its primary clinical application lies in excluding rather than identifying NAFLD. Further investigation is required to assess the performance of the suggested FSH cut-off values in identifying the high-risk individuals for NAFLD, particularly in the older population.

The current investigation possesses a number of limitations. Initially, as we conducted a retrospective observational study, it is not possible to establish the causative nature of the connections. Secondly, hepatic steatosis was determined using ultrasound imaging without the verification of a liver biopsy. Nevertheless, routinely performing a liver biopsy is not considered acceptable. Thirdly, while we analyzed the medical history of liver disease through self-report questionnaires, there is a potential for incomplete exclusion of rare liver conditions like autoimmune hepatitis and Wilson’s disease due to the inherent recall bias associated with self-reporting. Given the rarity of these diseases, this may not have significant implications on the study outcomes. Finally, it is worth noting that this study was exclusively carried out at a single medical facility situated in China. Consequently, caution must be exercised when generalizing the results to populations with more extensive racial and ethnic diversity. Although this study has its limitations, it makes a significant contribution by being the first to establish a connection between FSH and NAFLD in elderly individuals of both genders while accounting for all potential confounding factors. To validate the causality between NAFLD and FSH levels, there is a need for further multicenter longitudinal cohort studies. Additionally, well-designed randomized clinical trials are necessary to confirm the findings elucidated in this research.

## Conclusions

In conclusion, this study demonstrates an independent correlation between low FSH levels and NAFLD in aging population. The significance of this relationship remains even after accounting for all potential confounding factors. This research presents novel findings regarding the relationship between FSH and NAFLD, offering opportunities to explore innovative therapeutic targets for the management of this condition.

### Electronic supplementary material

Below is the link to the electronic supplementary material.

Supplementary Material 1

## Data Availability

The datasets used and/or analyzed during the current study can be obtained from the corresponding author on reasonable request.

## References

[1] Diehl AM, Day C (2017). Cause, Pathogenesis, and treatment of nonalcoholic steatohepatitis. N Engl J Med.

[2] Mir BA, Majeed T, Chauhan A (2022). Nonalcoholic fatty liver disease. N Engl J Med.

[3] Younossi Z, Tacke F, Arrese M (2019). Global perspectives on nonalcoholic fatty liver disease and nonalcoholic steatohepatitis. Hepatology.

[4] Sanyal AJ (2019). Past, present and future perspectives in nonalcoholic fatty liver disease. Nat Rev Gastroenterol Hepatol.

[5] Soderberg C, Stal P, Askling J (2010). Decreased survival of subjects with elevated liver function tests during a 28-year follow-up. Hepatology.

[6] Adams LA, Lymp JF, St Sauver J (2005). The natural history of nonalcoholic fatty liver disease: a population-based cohort study. Gastroenterology.

[7] Stefan N, Cusi K (2022). A global view of the interplay between non-alcoholic fatty liver disease and diabetes. Lancet Diabetes Endocrinol.

[8] Fabbrini E, Sullivan S, Klein S (2010). Obesity and nonalcoholic fatty liver disease: biochemical, metabolic, and clinical implications. Hepatology.

[9] Friedman SL, Neuschwander-Tetri BA, Rinella M, Sanyal AJ (2018). Mechanisms of NAFLD development and therapeutic strategies. Nat Med.

[10] Grossmann M, Wierman ME, Angus P, Handelsman DJ (2019). Reproductive Endocrinology of nonalcoholic fatty liver disease. Endocr Rev.

[11] Phan H, Richard A, Lazo M et al. The association of sex steroid hormone concentrations with non-alcoholic fatty liver disease and liver enzymes in US men. Liver Int. 2020. 10.1111/liv.14652 PMC10115140 32860311

[12] Sarkar M, Yates K, Suzuki A et al. Low testosterone is Associated with Nonalcoholic Steatohepatitis (NASH) and severity of NASH Fibrosis in Men with NAFLD. Clin Gastroenterol Hepatol. 2019. 10.1016/j.cgh.2019.11.053 PMC7272262 31812658

[13] Galmes-Pascual BM, Martinez-Cignoni MR, Moran-Costoya A (2020). 17beta-estradiol ameliorates lipotoxicity-induced hepatic mitochondrial oxidative stress and insulin resistance. Free Radic Biol Med.

[14] Landomiel F, Gallay N, Jegot G (2014). Biased signalling in follicle stimulating hormone action. Mol Cell Endocrinol.

[15] Cui H, Zhao G, Liu R, Zheng M, Chen J, Wen J (2012). FSH stimulates lipid biosynthesis in chicken adipose tissue by upregulating the expression of its receptor FSHR. J Lipid Res.

[16] Sun L, Peng Y, Sharrow AC (2006). FSH directly regulates bone mass. Cell.

[17] Mancinelli R, Onori P, Gaudio E (2009). Follicle-stimulating hormone increases cholangiocyte proliferation by an autocrine mechanism via cAMP-dependent phosphorylation of ERK1/2 and Elk-1. Am J Physiol Gastrointest Liver Physiol.

[18] Song Y, Wang ES, Xing LL (2016). Follicle-stimulating hormone induces postmenopausal Dyslipidemia through inhibiting hepatic cholesterol metabolism. J Clin Endocrinol Metab.

[19] Serviente C, Tuomainen TP, Virtanen J, Witkowski S, Niskanen L, Bertone-Johnson E (2019). Follicle-stimulating hormone is associated with lipids in postmenopausal women. Menopause.

[20] Wang N, Kuang L, Han B (2016). Follicle-stimulating hormone associates with prediabetes and diabetes in postmenopausal women. Acta Diabetol.

[21] Wang N, Li Q, Han B (2016). Follicle-stimulating hormone is associated with non-alcoholic fatty liver disease in chinese women over 55 years old. J Gastroenterol Hepatol.

[22] Zhu Y, Xu J, Zhang X, Ke Y, Fu G, Guo Q (2021). A low follicle-stimulating hormone level is a protective factor for non-alcoholic fatty liver disease in older men aged over 80. BMC Geriatr.

[23] Xu Y, Wang L, He J (2013). Prevalence and control of diabetes in chinese adults. JAMA.

[24] de Alwis NM, Day CP (2008). Non-alcoholic fatty liver disease: the mist gradually clears. J Hepatol.

[25] Chobanian AV, Bakris GL, Black HR (2003). Seventh report of the Joint National Committee on Prevention, detection, evaluation, and treatment of high blood pressure. Hypertension.

[26] American Diabetes A (2020). 2. Classification and diagnosis of diabetes: Standards of Medical Care in Diabetes-2020. Diabetes Care.

[27] Alberti KG, Eckel RH, Grundy SM, the International Diabetes Federation Task Force on Epidemiology and Prevention; National Heart, Lung, and Blood Institute; American Heart Association. Harmonizing the metabolic syndrome: a joint interim statement of; World Heart Federation; International Atherosclerosis Society; and International Association for the Study of Obesity. Circulation. 2009;120(16):1640-5. 10.1161/CIRCULATIONAHA.109.192644 19805654

[28] Bedogni G, Bellentani S, Miglioli L (2006). The fatty liver index: a simple and accurate predictor of hepatic steatosis in the general population. BMC Gastroenterol.

[29] Wang J, Qin T, Sun J, Li S, Cao L, Lu X (2022). Non-invasive methods to evaluate liver fibrosis in patients with non-alcoholic fatty liver disease. Front Physiol.

[30] Craven L, Rahman A, Nair Parvathy S (2020). Allogenic fecal microbiota transplantation in patients with nonalcoholic fatty liver Disease improves abnormal small intestinal permeability: a Randomized Control Trial. Am J Gastroenterol.

[31] Li X, Jing L, Lin F (2018). Diurnal rhythm of follicle-stimulating hormone is associated with nonalcoholic fatty liver disease in a chinese elderly population. Eur J Obstet Gynecol Reprod Biol.

[32] Ge S, Zheng Y, Du L et al. Association between follicle-stimulating hormone and nonalcoholic fatty liver disease in postmenopausal women with type 2 diabetes mellitus. J Diabetes. 2023. 10.1111/1753-0407.13394 PMC10415867 37221966

[33] Wide L, Naessen T, Sundstrom-Poromaa I, Eriksson K (2007). Sulfonation and sialylation of gonadotropins in women during the menstrual cycle, after menopause, and with polycystic ovarian syndrome and in men. J Clin Endocrinol Metab.

[34] Randolph JF, Zheng H, Sowers MR (2011). Change in follicle-stimulating hormone and estradiol across the menopausal transition: effect of age at the final menstrual period. J Clin Endocrinol Metab.

[35] Kim C, Randolph JF, Golden SH (2015). Weight loss increases follicle stimulating hormone in overweight postmenopausal women [corrected]. Obes (Silver Spring).

[36] Liu XM, Chan HC, Ding GL (2015). FSH regulates fat accumulation and redistribution in aging through the Galphai/Ca(2+)/CREB pathway. Aging Cell.

[37] Wang H, Bu S, Tang J, Li Y, Liu C, Dong J (2021). PTPN5 promotes follicle-stimulating hormone secretion through regulating intracellular calcium homeostasis. FASEB J.

[38] Qiao S, Alasmi S, Wyatt A (2023). Intra-pituitary follicle-stimulating hormone signaling regulates hepatic lipid metabolism in mice. Nat Commun.

[39] Reame NE, Wyman TL, Phillips DJ, de Kretser DM, Padmanabhan V (1998). Net increase in stimulatory input resulting from a decrease in inhibin B and an increase in activin A may contribute in part to the rise in follicular phase follicle-stimulating hormone of aging cycling women. J Clin Endocrinol Metab.

[40] Santoro N, Adel T, Skurnick JH (1999). Decreased inhibin tone and increased activin A secretion characterize reproductive aging in women. Fertil Steril.

[41] Andersen GO, Ueland T, Knudsen EC (2011). Activin A levels are associated with abnormal glucose regulation in patients with myocardial infarction: potential counteracting effects of activin A on inflammation. Diabetes.

