# Supplementary material for: Follicle-stimulating hormone is negatively associated with nonalcoholic fatty liver disease in a Chinese elderly population: a retrospective observational study
Source: BMC Endocr Disord. 2023 Aug 7;23:165. doi: 10.1186/s12902-023-01427-x (PMC10405433; doi:10.1186/s12902-023-01427-x)
Supplement: Supplementary file 1 — Supplementary Material 1 [file 12902_2023_1427_MOESM1_ESM.docx]

Supplement 1 Collinearity analysis of systolic blood pressure and diastolic blood pressure

|  | Variance inflation factor | Tolerance |
| --- | --- | --- |
| diastolic blood pressure | 1.0 | 1.0 |

Supplement 2 Collinearity analysis of alanine aminotransferase and aspartate aminotransferase

|  | Variance inflation factor | Tolerance |
| --- | --- | --- |
| aspartate aminotransferase | 1.0 | 1.0 |

Supplement 3 Collinearity analysis of total cholesterol and other lipids

|  | Variance inflation factor | Tolerance |
| --- | --- | --- |
| triglycerides | 1.238 | 0.808 |
| low-density lipoprotein cholesterol | 1.034 | 0.967 |
| high-density lipoprotein cholesterol | 1.269 | 0.788 |

Supplement 4 Collinearity analysis of follicle-stimulating hormone and estradiol, testosterone, age

|  | Variance inflation factor | Tolerance |
| --- | --- | --- |
| estradiol | 1.229 | 0.814 |
| testosterone | 1.242 | 0.805 |
| age | 1.082 | 0.924 |

Supplement 5 Logistic regression analysis of the relationship between NAFLD defined by LFI and FSH according to gender and age stratification

|  |  |  | adjusted association |  |
| --- | --- | --- | --- | --- |
|  |  | Unadjusted association | Model 1 | Model 2 |
| All | Odds ratios | 0.990 | 0.984 | 0.982 |
|  | 95%CI | 0.984-0.997 | 0.973-0.996 | 0.971-0.993 |
|  | P value | 0.003 | 0.010 | 0.001 |
| Men*  N=98 | Odds ratios | 0.983 | 0.961 | 0.957 |
|  | 95%CI | 0.957-1.010 | 0.923-1.000 | 0.919-0.997 |
|  | P value | 0.208 | 0.050 | 0.034 |
| Women*  N=357 | Odds ratios | 0.983 | 0.982 | 0.984 |
|  | 95%CI | 0.974-0.992 | 0.970-0.995 | 0.972-0.995 |
|  | P value | <0.001 | 0.007 | 0.004 |
| Age stratification |  |  |  |  |
| between 60-70 years | Odds ratios | 0.991 | 0.988 | 0.984 |
| N=230 | 95%CI | 0.983-0.999 | 0.972-1.003 | 0.970-0.999 |
|  | P value | 0.035 | 0..127 | 0.042 |
| Over 70 years | Odds ratios | 0.990 | 0.984 | 0.982 |
| N=225 | 95%CI | 0.984-0.997 | 0.973-0.996 | 0.971-0.993 |
|  | P value | 0.003 | 0.010 | 0.001 |

Model 1 included age, gender, body mass index, systolic blood pressure, fasting blood glucose, triglycerides, total cholesterol, high-density lipoprotein cholesterol, low-density lipoprotein cholesterol, alanine aminotransferase, aspartate aminotransferase, estradiol and testosterone for adjustment.

Model 2 included age, gender, alanine aminotransferase, aspartate aminotransferase, estradiol, testosterone, diabetes mellitus, dyslipidemia, obesity and hypertension for adjustment.

NAFLD: nonalcoholic fatty liver disease; FSH: follicle-stimulating hormone.

*: All variables except gender were included in the models.
